# Reply to: ‘Childhood leukaemia and socioeconomic status in England and Wales 1976–2005: evidence of higher incidence in relatively affluent communities persists over time’

**DOI:** 10.1038/bjc.2012.180

**Published:** 2012-06-12

**Authors:** M E Kroll, C A Stiller, M F G Murphy

**Affiliations:** 1 Childhood Cancer Research Group, Department of Paediatrics, University of Oxford, Richards Building, Oxford OX3 7LG, UK; 2 Cancer Epidemiology Unit, Nuffield Department of Clinical Medicine, University of Oxford, Richard Doll Building, Oxford OX3 7LF, UK

**Sir,**

We thank you for giving us the opportunity to reply to the letter from Dr Smith *et al* (2012).

Using records from the National Registry of Childhood Tumours for England and Wales 1976–2005, we found a statistically significant decreasing trend in reported incidence of lymphoid leukaemia with increasing deprivation, based on residence at diagnosis (Kroll *et al*, 2011). In children, lymphoid leukaemia consists almost entirely of acute lymphoblastic leukaemia (ALL). Dr Smith *et al* (2012) suggest that we inappropriately selected results from their publication to support our findings. In our view, the cited results formed part of a summary of existing research on this topic, and were selected for relevance and validity.

Selection was necessary because Smith *et al* (2006) presented eight different analyses relating ALL to socioeconomic status, four of which used a deprivation measure based on residence at diagnosis (a continuous score ranging from −6.15 to +7.75). Only one of these analyses (A in the Table accompanying this letter) followed the methods that would normally be considered appropriate for an individually matched case–control study. The other three took a more unconventional approach. Smith *et al* (2006) chose to quote the results of one of these alternatives (B) in the abstract of their paper. For comparison with our study we prefer A, because we are confident that this analysis was valid. The findings of A and B are different.

In their letter, Dr Smith *et al* (2012) state that ‘non-interviewed cases tended to live in more-deprived areas’, and that analysis B was preferred on the grounds that ‘these results were based on all cases diagnosed across the country as a whole and all randomly selected ‘first-choice’ controls – regardless of whether or not their parents were interviewed in the main study’. However, the controls were matched to interviewed cases only. We would argue that, through the matching on a large number of districts of residence (113 geographical areas), the study is likely to have been partially matched on socioeconomic status. If so, the controls, like the interviewed cases they were matched to, would have been deficient in children from poorer communities. This would imply that when the cases were extended to include non-interviewed cases, as in analysis B, there would be a breach of the requirement that cases and controls are selected from the same underlying population. We are also concerned about the extent to which the adjustment factors used in the unmatched analysis would compensate for breaking the matching.

In an earlier analysis of the same case–control study, Law *et al* (2003) found a significant association of ALL risk with deprivation, using conventional methods. The odds ratios presented in the table of Law *et al* (2003) suggest a decreasing trend with increasing deprivation, consistent with analysis A above.

Smith *et al* (2006) attributed the socioeconomic gradient seen in other studies to artefact, and implied that case ascertainment for their study was more complete than the national childhood cancer registration system. In fact, the reverse appears to be true. For Great Britain, in the 2 years during which the study aimed for national coverage of all childhood cancers (1993–1994), 2650 cases were registered, of which 722 were ALL (Smith *et al*, 2006). These counts are, respectively, 10% and 2% less than the corresponding figures from the National Registry of Childhood Tumours, which were 2955 cases in total (International Classification of Childhood Cancer, third edition, groups I–XII), of which 739 were ALL.

We consider that the socioeconomic gradient in recorded incidence of childhood leukaemia is interesting and important for epidemiological reasons, and perhaps also for clinical reasons. A further study (Kroll *et al*, 2012) uses clinical data to investigate the possibility that under-diagnosis of childhood ALL in poorer communities might be a contributing factor.

## Figures and Tables

**Table:** Summary of two analyses relating risk of childhood ALL to a deprivation measure based on residence at diagnosis (Source: Tables 1 and 3 in Smith *et al*, 2006)

	**Odds ratio (95% confidence interval)**	
	**Analysis A**	**Analysis B**
Cases	Interviewed ALL cases (*N*=1460)	Registered ALL cases, including non-interviewed cases (*N*=1578)
Controls	Controls matched to interviewed ALL cases (*N*=2919)	Controls matched to interviewed cases of any type of childhood cancer (*N*=7663)
Matching factors	Birthdate, sex, 113 geographical areas	Birthdate, sex, 113 geographical areas
Adjustment factors	Not applicable	Age at diagnosis, sex, 10 regions
Analysis	Matched	Unmatched
		
*Deprivation fifths*		
1 Affluent	1.00	1.00
2	1.10 (0.90–1.34)	1.10 (0.93–1.30)
3	0.89 (0.73–1.09)	0.96 (0.81–1.14)
4	0.97 (0.79–1.19)	1.02 (0.86–1.22)
5 Deprived	0.76 (0.61–0.95)	0.90 (0.75–1.07)
		
Trend per unit of the continuous deprivation score	0.96 (0.94–0.99) i.e., statistically significant	0.99 (0.96–1.01) i.e., not statistically significant

Abbreviation: ALL=acute lymphoblastic leukaemia.
